# Activation of Microbiota Sensing – Free Fatty Acid Receptor 2 Signaling Ameliorates Amyloid-β Induced Neurotoxicity by Modulating Proteolysis-Senescence Axis

**DOI:** 10.3389/fnagi.2021.735933

**Published:** 2021-10-05

**Authors:** Atefeh Razazan, Prashantha Karunakar, Sidharth P. Mishra, Shailesh Sharma, Brandi Miller, Shalini Jain, Hariom Yadav

**Affiliations:** ^1^Department of Internal Medicine, Molecular Medicine, Wake Forest School of Medicine, Winston Salem, NC, United States; ^2^Department of Biotechnology, PES University, Bangalore, India; ^3^Department of Neurosurgery and Brain Repair, Morsani College of Medicine, University of South Florida, Tampa, FL, United States; ^4^National Institute of Animal Biotechnology, Hyderabad, India; ^5^Department of Internal Medicine—Digestive Diseases and Nutrition, Morsani College of Medicine, University of South Florida, Tampa, FL, United States; ^6^USF Center for Microbiome Research, USF Institute on Microbiomes, Center of Excellence for Aging and Brain Repair, University of South Florida, Tampa, FL, United States

**Keywords:** microbiota, free fatty acid, G-coupled protein receptor, FFAR2 (GPR43), fenchol, natural compounds, Alzheimer’s

## Abstract

Multiple emerging evidence indicates that the gut microbiota contributes to the pathology of Alzheimer’s disease (AD)—a debilitating public health problem in older adults. However, strategies to beneficially modulate gut microbiota and its sensing signaling pathways remain largely unknown. Here, we screened, validated, and established the agonists of free fatty acid receptor 2 (FFAR2) signaling, which senses beneficial signals from short chain fatty acids (SCFAs) produced by microbiota. The abundance of SCFAs, is often low in the gut of older adults with AD. We demonstrated that inhibition of FFAR2 signaling increases amyloid-beta (Aβ) stimulated neuronal toxicity. Thus, we screened FFAR2 agonists using an in-silico library of more than 144,000 natural compounds and selected 15 of them based on binding with FFAR2-agonist active sites. Fenchol (a natural compound commonly present in basil) was recognized as a potential FFAR2 stimulator in neuronal cells and demonstrated protective effects against Aβ-stimulated neurodegeneration in an FFAR2-dependent manner. In addition, Fenchol reduced AD-like phenotypes, such as Aβ-accumulation, and impaired chemotaxis behavior in *Caenorhabditis (C.) elegans* and mice models, by increasing Aβ-clearance *via* the promotion of proteolysis and reduced senescence in neuronal cells. These results suggest that the inhibition of FFAR2 signaling promotes Aβ-induced neurodegeneration, while the activation of FFAR2 by Fenchol ameliorates these abnormalities by promoting proteolytic Aβ-clearance and reducing cellular senescence. Thus, stimulation of FFAR2 signaling by Fenchol as a natural compound can be a therapeutic approach to ameliorate AD pathology.

## Introduction

Alzheimer’s disease (AD) is the most common neurodegenerative disorder in older adults(Jonsson et al., 2013). Its prevalence is increasing, and no successful preventative therapies or treatments are available. AD is commonly characterized by a higher accumulation of amyloid-beta (Aβ) and the formation of intracellular neurofibrillary tangles, which leads to neuronal death and a decline in memory and learning behaviors (Singh et al., 2016; De Ture and Dickson, 2019). Although mechanisms underlying increased Aβ accumulation in the brain (an important hallmark of AD) are not known, it increases neurotoxicity/neuronal death (neurodegeneration) and impairs learning and memory behaviors (Yankner, 1996; Selkoe, 2001; De Ture and Dickson, 2019). Current therapies that are targeted to reduce Aβ levels are not successful, but there is a plethora of literature suggesting that an increased Aβ accumulation is causal for neurodegeneration in AD pathology. Thus, reducing their accumulation can be an effective strategy to prevent AD progression. Increased accumulation of Aβ is associated with either higher production or reduced clearance (Liu et al., 2016). A reduced clearance is a hallmark of Aβ accumulation and is linked to reduced proteasomal/lysosomal activities in the brain (Buoso et al., 2012; Settembre et al., 2013; Vilchez et al., 2014; Leeman et al., 2018). Increased Aβ accumulation is known to stimulate senescence in neuronal and microglial cells, which initiates a cascade of detrimental events, including neuroinflammation (Heneka et al., 2015). Inflammatory cytokines instigate Aβ production by activating specific transcription factors, such as interferon-induced transmembrane protein three in neurons and astrocytes (Hur et al., 2020). Together, these abnormalities further deteriorate brain health and functions, such as memory and learning behaviors (He et al., 2013). However, because we do not fully understand the pathology of Aβ production and its accumulation and clearance, strategies to reduce the accumulation of Aβ in the neuronal cells and, therefore, ameliorate neurodegeneration and AD pathology, are lacking.

Multiple emerging evidence, including our recent studies, demonstrated that the gut microbiota is significantly different in patients with mild cognitive impairment (MCI- an early stage of AD) and AD (Nagpal et al., 2019, 2020; Doifode et al., 2021). Indeed, fecal microbiota transplantation (FMT) from AD donor animals increases the progression of AD pathology in recipient mice, while FMT of healthy donors decreases its progression (Sun et al., 2019), suggesting that the gut microbiota abnormalities are causal in AD pathology and their restoration can ameliorate AD progression. Although the mechanisms by which the gut microbiota contributes to brain health remain largely unknown, emerging data show that microbial metabolites are a possible link (Vogt et al., 2018; Fujii et al., 2019; Konjevod et al., 2020; Zhuang et al., 2021; Doifode et al., 2021). For example, short chain fatty acids (SCFAs, such as acetate, propionate, and butyrate) are the major beneficial metabolites produced by healthy microbiota; they exhibit beneficial effects on the brain, including a reduction in AD pathology, in several animal models (Mishra et al., 2020; Sun et al., 2020; Wenzel et al., 2020). Indeed, the abundance of SCFAs is often reduced in the gut of patients with AD and MCI, while healthy dietary habits, like a ketogenic diet, increase their production (Nagpal et al., 2019, 2020). However, the mechanisms by which a decrease in SCFAs contributes to AD progression and repletion of SCFAs protects from AD pathology remain largely unknown. Herein, we demonstrated that suppressing free fatty acid receptor 2 (FFAR2; a G-protein-coupled receptor activated by SCFAs) contributes to Aβ accumulation and discovered that Fenchol, a natural compound, decreased AD pathology by activating FFAR2 signaling.

## Materials and Methods

### Cell Culture and FFAR2 Expression in Cells

Human neuroblastoma cells-SK-N-SH (HTB-11; American Type Culture Collection, Manassas, VA, USA) and HEK293 cells (as positive control) were cultured in DMEM medium supplemented with 10% FBS and 1% antibiotics (penicillin and streptomycin), grown at 37°C and subcultured every 48 h. We measured FFAR2 expression in these cells by harvesting total RNA while cells were at 70–80% confluency, using the RNAeasy kit (Qiagen). The expression of FFAR2 mRNA was quantified using real-time PCR after converting total RNA to cDNA using the High-Capacity cDNA Reverse Transcription kit (Applied Biosystems). 18S RNA was used as an internal control. The fold change in gene expression was determined using delta-delta Ct values, following our routinely used well-established and published protocols (Nagpal et al., 2018; Ahmadi et al., 2019, 2020a,b; Wang et al., 2020).

### FFAR2 Inhibition and Aβ-Induced Neurotoxicity Using MTT Assay

To determine the impact of FFAR2 inhibition on Aβ-induced neuronal cell toxicity, we performed the MTT viability assay (Ronicke et al., 2008). SK-N-SH cells (1 × 10^3^ cells per well) were plated in a 96-well plate containing DMEM medium supplemented with 10% FBS and 1% antibiotics and grown at 37°C for 24 h. Then, media was removed and cells were washed with PBS before being replenished with DMEM media containing 1% FBS and 10 μM FFAR2 inhibitor (CATPB, Tocris) and incubated at 37°C for 2 h. Then, cells were treated with Aβ 25–32 peptide (25 μM, Sigma) and incubated for 4 h. Afterward, the media was replaced with 100 μl of fresh media, and 10 μl of MTT reagent (5 mg/ml in phosphate buffer [pH 7.2]) was added and mixed in each well before an additional 4 h incubation. Then, DMSO (50 μl) was added in each well and mixed thoroughly to dissolve formazan crystals. The absorbance was measured at 570 nm.

### In-silico Screening of Natural FFAR2 Activators

The primary protein sequences of human and mouse FFAR2 were retrieved from the Uniprot database in FASTA format with the Uniprot IDs O15552 and Q8VCK6, respectively. The structures of 144,856 natural compounds were obtained from ZINC database (Supplementary Table S1). Through in-silico virtual screening of these compounds based on binding to human FFAR2 using AutoDock6 software, we shortlisted the top 15 compounds (Supplementary Table S2). Furthermore, the tertiary structures of both mouse FFAR2 and human FFAR2 were predicted using the threading-based online server I-TASSER (Zhang, 2008; Roy et al., 2010; Yang et al., 2015), refined using ModRefiner (Xu and Zhang, 2011), and validated using the Ramachandran plot from RAMPAGE server (Lovell et al., 2003). The natural activators, including acetate, butyrate, and the shortlisted compounds, were blindly docked with both mouse FFAR2 and human FFAR2 using AutoDock Vina to find the compounds that demonstrated activator properties (Trott and Olson, 2010). AutoGrid program was used to fix the grid box around the proteins. The grid box for the human FFAR2 binding site was set to the XYZ coordinates of 68.028, 66.787, and 54.804, respectively. The box dimensions were 28.089, 28.562, and 25.349 along the X, Y, and Z axes, respectively. For the mouse FFAR2, the grid box binding site was set to XYZ coordinates of 63.359, 66.205, and 46.924, and the box dimensions were 40.229, 41.757, and 36.283, respectively. The nine conformations along with the Vina score were used to screen the activator. The interaction of the ligands was analyzed using the 2D interaction diagrams generated by using LigPlot+ (Laskowski and Swindells, 2011) and 3D interaction images using PyMOL2.3 (The PyMOL Molecular Graphics System, Version 2.0 Schrödinger, LLC).

### Western Blotting

To determine the FFAR2 signal activating property of selected compounds, we treated SK-N-SH cells with 10 μM of shortlisted compounds for 30 min at 37°C and harvested for total protein extraction. We harvested cells after 30 min of incubation because ERK1/2 phosphorylation upon FFAR2 activation is a quick event (Jiao et al., 2002; Bhattacharjee et al., 2010). Total protein from the cells was extracted using a lysis buffer [consisting of 10 mmol/L Tris (pH 7.6), 150 mmol/L NaCl, 10 mmol/L Sodium orthovanadate, 10 mmol/L Sodium Pyrophosphate, 100 mmol/L Sodium fluoride, 1 mmol/L EDTA, 1 mmol/L EGTA, 1% Triton X-100, 0.5% NP-40 and a cocktail of protease inhibitors (Roche); Yadav et al., 2011, 2017; Ahmadi et al., 2019, 2020a]. After sonication, cell extracts were centrifuged at 8,000× *g* for 10 min at 4°C, and the supernatant was separated in a fresh tube. The total protein concentration of the supernatant was measured using a BCA protein assay kit. An equal amount of total protein (45 μg) from each sample was dissolved in loading buffer and loaded in 4% stacking gel and 10% separating SDS-PAGE gel and resolved at 100 V. Proteins were transferred on a 0.22 μm PVDF membrane and blots were developed with anti-ERK1/2, anti-phosphoERK1/2, ubiquitin (P4D1), and Aβ (6E10) primary antibodies followed by their secondary antibodies and bands were visualized using the Pierce™ Fast Western Blot Kit, ECL Substrate acquired using Syngene Pxi. Band densities were also quantified using NIH ImageJ software. β-actin was used as an internal control.

### Cyclic Adenosine Monophosphate (cAMP) Assay

FFAR2 is a Gi/o signaling receptor, which reduces cytosolic cAMP (Falomir-Lockhart et al., 2019). To determine the FFAR2 signaling activating potential of selected compounds, we used the cAMP-Glo™ Assay to measure intracellular cAMP. In brief, SK-N-SH cells (1 × 10^3^ cells per well) were plated in a white 96-well plate for 24 h and then treated with 10 μM compounds and 10 μM forskolin (a positive control) dissolved in induction buffer. The cAMP levels were detected using a Luminescence Microplate Reader.

### Intracellular Calcium Assay

FFAR2 signaling activation increases intracellular calcium (Falomir-Lockhart et al., 2019). To further determine the activation potential of selected compounds for this signaling, we plated the SK-N-SH cells (1.25 × 10^3^ cells per well) into a dark 96-well plate for 24 h. Then cells were treated with 10 μM selected compound(s) and 10 mM acetate (an SCFA positive control). Also, 0.01 M EDTA (Sigma) and 114 nm carbachol were used as negative and additional positive controls, respectively. After 15 min, the cells were treated with 2× fluo-4 Direct™ calcium reagent along with 250 mM probenecid and further incubated at 37 °C for 30 min. The fluorescence was measured with excitation at 494 nm and emission at 516 nm using a microplate reader.

### Culture and Maintenance of ***C. elegans***

We have used *C. elegans* as a model for determining the impact of selected compounds on aging and AD-like phenotypes. The wild-type (N2) and CL2122 (dvls15 [9pPD30.38) unc-54(vector) + (pCL26)mtl-2::GFP]) were propagated at 20°C. AD transgenic worms model CL2006 (dvls [pCL12(unc-54/human abeta peptide 1–42 minigene) + pRF4), CL4176 (dvls27[myo-3p::A-Beta (1–42)::let-851 3′UTR) + rol-6 (su1006] and CL2355 (dvls 50 [pCL45 9snb-1::Abeta 1–42::3′ UTR 9 log) + mtl-2::GFP]) were maintained at 16°C. All strains were cultured on solid nematode growth medium (NGM) consisting of 3 g/L NaCl, 2.5 g/L peptone, 5 mg/L cholesterol, 1 mM CaCl2, 1 mM MgSO_4_, 25 mM KH_2_PO_4_, and 17 g/L agar and seeded with OP50 (*Escherichia coli* strain) as a food supply. The worms were allowed to grow up to the adult stage, and egg carrying worms were used to isolate age-synchronized eggs using the bleaching method (Porta-de-la-Riva et al., 2012). The synchronized eggs were cultured on an NGM plate or fresh S-Complete plus media with 5% OP50, and 10 μM Fenchol (Sigma) at either 20°C (N2 and CL2122) or 16°C (CL2006, CL4176, and CL2355). All of the strains used in this study were acquired from the University of Minnesota- Caenorhabditis Genetics Center, which is funded by the NIH Office of Research Infrastructure Programs (P40 OD010440).

### Lifespan and Paralysis Assays in ***C. elegans***

To determine the impact of selected compounds on age-related decline in brain health, we first measured the lifespan of the worms. The L1 worms were cultured in 96-well plates on S-complete media containing *E. coli* OP50 and antibiotics (about 10–15 animals per well). The experimental group worms were treated with 10 μM Fenchol and control groups were treated with 0.1% DMSO. The 0.6 mM Fluorodeoxyuridine (FUDR; 30 μl) was added to each well after 3 days and animals were monitored every other day for viability. The CL4176 and CL2006 worms were grown at 16 °C and N2 worms were grown at 20°C.

For paralysis assays, synchronized eggs of CL4176 were kept at 16°C for 48 h, and the worms were transferred onto NGM plates. Experimental group worms were treated with 10 μM Fenchol and control groups were treated with DMSO at 25 °C for 20 h. Counting was performed at 2 h intervals, typically after 14 h, at 23°C, and until the last worm became paralyzed. If worms did not move or only moved their head but not their body when gently touched with a platinum loop, they were scored as paralyzed. Each *C. elegans* experiment was conducted with at least 100 worms.

### Thioflavin-S-Staining

To detect amyloid plaques in the transgenic CL2006 worms, age-synchronized worms were grown on media with and without Fenchol supplementation for up to 5 days of age and then worms were fixed in 4% paraformaldehyde- PBS. Then, the worm bodies were permeabilized in 1% Triton X-100, 125 mM Tris (pH 7.4), and 5% fresh β-mercaptoethanol. After washing three times with 1% PBS-Tween-20, the worm bodies were stained with 0.125% thioflavin S in 50% ethanol for 2 min. After washing three times with ethanol in the darkroom, worms were mounted on slides for microscopy using a Zeiss 710 confocal microscope equipped with a digital camera. A total of 20 worms were counted from each group.

### Measurement of Chemotaxis Activity

Both *C. elegans* strains (CL2122 and CL2355) were grown on media supplemented with 10 μg/ml Fenchol and DMSO (control) and OP50 lawn at 16 °C for 36 h, and then at 23 °C for another 36 h. Then the worms were washed twice with S-Basal media and transferred to an NGM plate without *E. coli* OP50. These plates were divided into four equal sectors, with 1 μl of 0.1% benzaldehyde as an odorant and 1 μl of 1 M sodium azide as an anesthetic to arrest the worms upon reaching the quadrant were added to the original spot. On the opposite side of the attractant, a 1 μl drop of sodium azide and 1 μl of control odorant (100% ethanol) were added onto the NGM agar plate. Around 40–45 worms were placed in the center of each plate, incubated at room temperature for 1 h, and transferred to 4°C overnight. The chemotaxis index (CI) was scored using the following formula: (number of worms at the attractant location − number of worms at the control location)/total number of worms on the plate (Margie et al., 2013).

### Mice Studies

To determine the impact of Fenchol treatment on the AD mice model, we treated 3 months old male APP/PS1 mice (Jackson Laboratory- Stock number: MMRRC Stock No: 34829-JAX) with Fenchol (80 mg/kg body weight dose by daily oral gavage) for 3 months and compared them to their DMSO treated controls. Brains were collected and immediately homogenized in proteasome lysis buffer and proteasome activity was measured as described below. All the animal procedures and experiments were approved by the institutional animal care and use committee (IACUC) of the Wake Forest School of Medicine and the University of South Florida.

### Proteasome Activity Assay

The CL2006 strain, SK-N-SH cells, and brain tissues were sonicated in a lysis buffer (150 mM NaCl, 50 mM HEPES, 2 mM DTT, 20 mM NaF, 5 mM EDTA, 2.5 mM sodium pyrophosphate, 1 mM sodium orthovanadate, and 1 mM α-glycerophosphoric acid) and then centrifuged at 15,000× *g* at 4 °C for 15 min. Total protein was assayed using BCA kit and 20 μg protein was mixed with 140 μM suc-leu-leu-val-tyr–7-amino-4-methylcou-marin (AMC) in a proteasome activity assay buffer (150 mM NaCl, 50 mM HEPES [pH 7.4], 5 mM ATP, and 5 mM EDTA), and then the fluorescence (excitation 380 nm and emission 460 nm) was measured every 10 min over 3 h using a microplate reader. The assay was performed with and without the proteasome inhibitor MG132 (10 μM).

### Lysosome Activity

The lysosomal activity was measured in 1 × 10^3^ SK-N-SH cells per well plated into a 96-well plate and treated with 10 μM Fenchol, 25 μm Aβ 25-32, 1% DMSO, and 500 nm bafilomycin. Bafilomycin was used as an inhibitor of cellular autophagy/lysosomal activity. The lysosomal activity was assayed using the Enz^®^ lysozyme assay kit (E-22013, Molecular Probes) and the fluorescence was measured in a fluorescence microplate reader using excitation and emission wavelengths of 494 nm and 518 nm, respectively (Biotek Instruments, VT, USA).

### Measurements of Senescence

Cells were treated with 10 μl Fenchol and DMSO. After overnight incubation, Aβ 25-32 (25 μM) peptide was added to the cells for 4 h. The Senescence β-Galactosidase Staining Kit (Cell Signaling, USA) was used for the detection of the expression of β-galactosidase activity, following the manufacturer’s instructions.

### Statistical Analyses

The Student t-test and one/two way ANOVA have been used to determine the statistical significance, as appropriate. All the assays were done in triplicate and repeated two-three times, and values were presented as the mean and standard error of means. P-values less than 0.05 were considered statistically significant.

## Results

### FFAR2 Is Expressed on Neuronal Cells and Its Inhibition Increases Aβ-Induced Neurotoxicity

**Figure 1:**
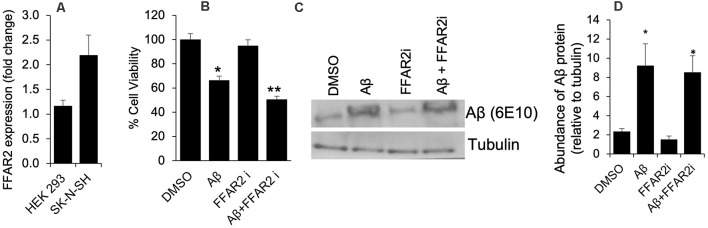
FFAR2 expressed in human neuronal cells and its inhibition increased Aβ-induced neurotoxicity. **(A)** The mRNA expression of FFAR2 gene in SK-N-SH and HEK293 cells. **(B)** SK-N-SH cells treated with FFAR2i (10 μM CATPB) for 4 h and Aβ25–35 (25 μM) for 2 h, then measured cell viability using MTT assay, show that Aβ-treatment induces cell death and inhibition of FFAR2 further reduced cell viability. DMSO used as vehicle control. **(C,D)** Similarly, Aβ-treatment significantly increased Aβ-accumulation in both with and without FFAR2 inhibition. Data are the mean (*n* = 3 replicates in three repeated experiments) ± SEM. *P* values with *<0.05 and **<0. 01 are significantly different.

To determine the role of FFAR2 in neurons, we first confirmed and found that the FFAR2 gene is abundantly expressed in human neuronal SK-N-SH cells, similar to HEK293 cells, which were used as a positive control (Figure 1A). As expected, we observed that Aβ- treatment significantly induced cell death in SK-N-SH cells (Figure 1B). However, the inhibition of FFAR2 using a small compound inhibitor (CATPB) further exacerbated Aβ-induced cell death in these cells (Figures 1C,D). These results indicate that FFAR2 is expressed in the neuronal cells and its inhibition exacerbates the detrimental effects of increased Aβ and decreases the survival of neuronal cells, suggesting that the activation of FFAR2 signaling may reduce Aβ-induced neuronal toxicity.

### Large-Scale Screening of Natural Compounds Demonstrated Fenchol as a Potential Activator of FFAR2 Signaling

**Figure 2:**
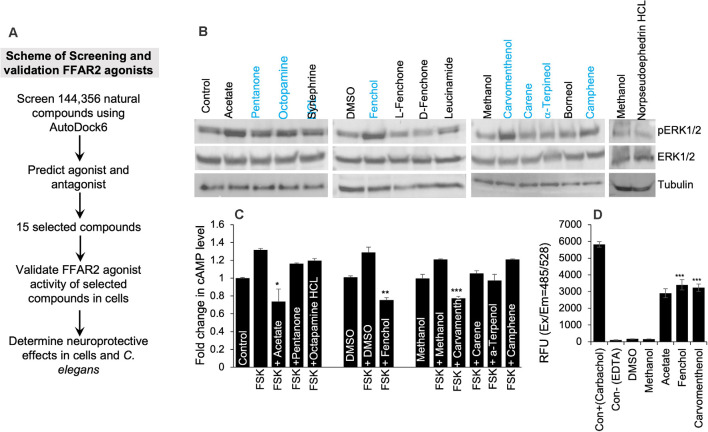
*In silico* and *in vitro* screening of FFAR2 activators discovered Fenchol as the most effective activator of FFAR2 signaling. **(A)** Schematic of screening FFAR2 activators and their validation for ameliorating AD-like phenotype. **(B)** FFAR2 activating potential of selected compounds in terms of increasing phospho-ERK1/2 levels. **(C)** Further selected compounds (*n* = 7) were showing a reduction in Forskolin (FSK) induced cAMP in SK-N-SH cells. **(D)** In addition, further selected (*n* = 2) for increasing intracellular calcium- another indication of FFAR2 signaling activation. Data are shown as the mean ± S.D. (*n* = 3 experiments in triplicate). *P < 0.05; **P < 0.01; ***P < 0.001.

Based on the in-silico screening of >144,000 natural compounds and their binding affinity to the active site of human FFAR2, we shortlisted the top 15 compounds (Figure 2A, Supplementary Table S2). To determine the interactions of these selected natural compounds and predict their activator vs. inhibitory properties to FFAR2, their interaction profiles were compared with known FFAR2 activators, like acetate and butyrate (Supplementary Tables S3A,B). The binding site residues Ser86, Tyr90, His140, Ile145, Val179, Arg180, Leu183, Tyr238, His242, and Arg255 were identified, similar to the published literature (Schmidt et al., 2011). We observed that acetate forms an H-bond with His242 in human FFAR2 and Arg65, Trp75, Tyr90, Gln148, Tyr238 in mouse FFAR2. Further, butyrate forms an H-bond with His242 in human FFAR2 and Arg65, Trp75, Tyr90, Gln148, Tyr238, and Arg255 in mouse FFAR2. Based on these (modeling) criteria, we classified the selected 15 compounds are potential ligands/activators for FFAR2.

In addition, to determine the FFAR2 activator activity of the selected 15 compounds in SK-N-SH cells, we used three levels of biological system screening based on measuring phospho-ERK1/2 and intracellular levels of cyclic AMP and calcium. Since FFAR2 activation increases phosphorylation of tyrosine amino acid of ERK1/2 (Liu et al., 2008), we used this as a *first-level biological screening*; and found a descending order of phopho-ERK1/2 levels as follows: Fenchol > Octopamine > Carvamenthenol > Camphene > Pentanone > Synephrine > Leucinamide > Carene > α-Terpineol > Breneol > L-Fenchone > D-Fenchone > Norpseudoephedrine HCl (Figure 2B). The levels of phospho-ERK1/2 proteins were highest in Fenchol and comparable to acetate, a known FFAR2 activator, in comparison to the rest of the selected compounds tested here (Figure 2B, Supplementary Figure S2B). Because FFAR2 activation decreases intracellular cAMP (Falomir-Lockhart et al., 2019) and increases calcium, we also verified and shortlisted our selected compounds to stimulate FFAR2 signaling by measuring intracellular cAMP (Falomir-Lockhart et al., 2019) and intracellular calcium levels (Al Mahri et al., 2020). Interestingly, Fenchol showed the highest decrease in cAMP in SK-N-SH cells upon Forskolin-stimulation, and such decreases were shown as Fenchol < Carvamenthenol < α-Terpineol < Carene < Pentanone < Octopamine < Camphene, in ascending order (Figure 2C). We used Forskolin as an inducer of an intracellular rise in cAMP levels (Curtin et al., 2006). Fenchol also showed the highest increase in intracellular calcium levels in SK-N-SH cells (Figure 2D). These results suggest that Fenchol is a potent activator of FFAR2 signaling and is conferred by an increase in phopho-ERK1/2 and calcium levels and a suppression of cAMP pathways.

**Figure 3:**
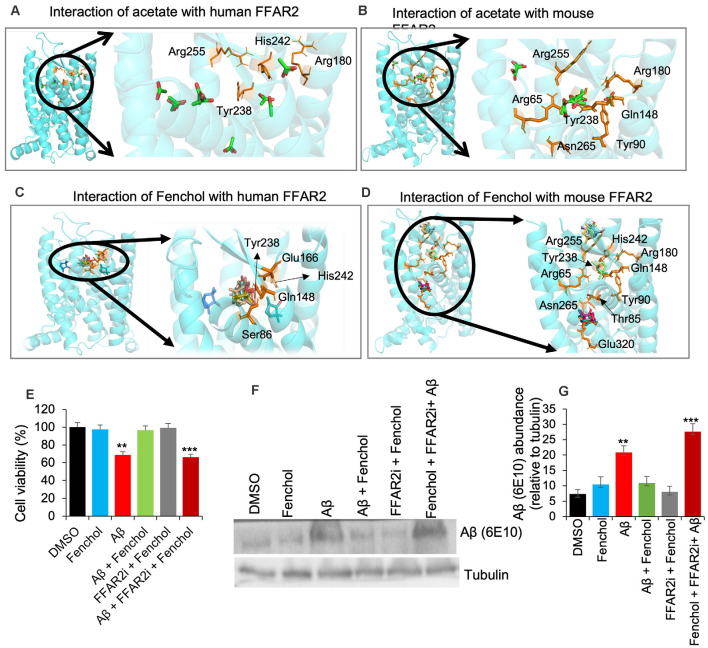
Fenchol binds to the FFAR2 active site and its neuroprotective effects are FFAR2 signaling dependent. **(A–D)** Interactions of Fenchol with human **(C)** and mouse **(D)** FFAR2 and its comparison with known FFAR2 agonist-Acetate **(A,B)**. **(E)** Fenchol treatment significantly reduced the Aβ-induced decline in cell viability **(E)** and Aβ-accumulation **(F,G)** in SK-N-SH cells on FFAR2 dependent manner. Data are mean ± SEM (*n* = 3 independent experiments in triplicate). **P < 0.01; ***P < 0.001.

Our further bioinformatics analyses revealed that the Fenchol (ZINC01081099) forms H-bonds with the Ser86, Gln148, Glu166, Tyr238, and His242 residues of human FFAR2 and the Thr85 and Glu320 residues of mouse FFAR2; these binding patterns are similar to those of acetate. The lowest Vina scores of −3 kcal/mol, −3.2 kcal/mol and −4.3 kcal/mol, −3.8 kcal/mol were obtained for acetate and butyrate of human and mouse FFAR2, respectively, while Fenchol also showed the lowest Vina scores of −5.5 kcal/mol and −5.3 kcal/mol with human FFAR2 and mouse FFAR2, respectively (Figures 3A–D). Further, molecular modeling analyses indicated that Fenchol and acetate have similar binding to human and mouse FFAR2 (Figures 3A–D), suggesting that Fenchol binds to the active site to stimulate FFAR2 signaling.

### Fenchol Protects Aβ-Induced Neurotoxicity in SK-N-SH Cells and *C. elegans*

Since the inhibition of FFAR2 exacerbates Aβ-induced neurotoxicity, we also determined the effects of Fenchol treatment against Aβ-induced SK-N-SH neuronal cell death. We show that the Fenchol treatment significantly reduced the Aβ-induced neuronal cell death in SK-N-SH cells compared to their DMSO controls during the MTT assay (Figure 3E). Interestingly, treatment with FFAR2 inhibitor and Fenchol demonstrated no significant protection in cell viability, indicating that the protective effects of Fenchol in Aβ-induced cell neurotoxicity are dependent on the activation of FFAR2 signaling. We also observed that Fenchol treatment significantly reduced Aβ-accumulation in SK-N-SH cells compared to their Aβ-only treated controls, again in an FFAR2 dependent manner (Figures 3F,G). This finding suggests that Fenchol treatment reduced Aβ-accumulation in neuronal cells by activating FFAR2 signaling, thus preventing cell death in the SK-N-SH cell culture system. However, the physiological importance of Fenchol in Aβ-accumulation and the underlying cellular and molecular mechanisms are still not known.

**Figure 4:**
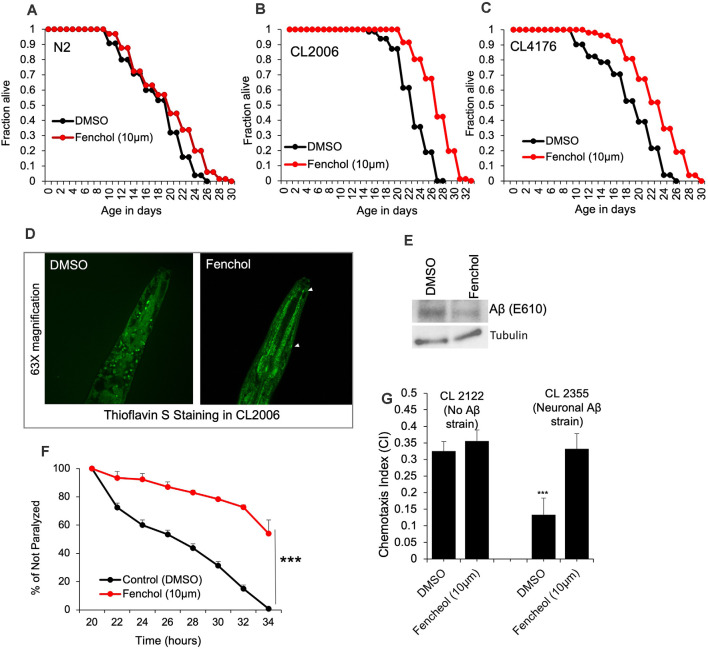
Fenchol significantly increased lifespan in human Aβ-over-expressing *C. elegans*. **(A–C)** Lifespan assay of wild-type **(A)** and human Aβ-overexpressing (CL2006 and CL4176) **(B,C)** with and without Fenchol treatments. **(D,E)** Aβ accumulation shown by thioflavin S staining **(D)** and western blot **(E)** in CL2006 worms treated with Fenchol in comparison to their DMSO treated controls. **(F)** Fenchol protected temperature-induced Aβ-mediated paralysis in CL4176 strains compared to their DMSO treated control group. **(G)** In addition, Fenchol treatment preserved temperature-induced Aβ overexpression mediated chemotaxis index (a markers of cognition/ memory) in CL2355 strain of *C. elegans* compared to their DMSO treated control, while Fenchol show normal effects in control CL2122 strain of *C. elegans*. Data are mean ± SEM (*n* = 3 independent experiments in triplicate). ***P < 0.001.

To demonstrate the physiological importance of Fenchol activity in reducing Aβ-induced neurotoxicity, we used *C. elegans* as a model of AD. Aging is one of the major risk factors for AD pathology and an increased risk of Aβ-induced neuronal cell death (Guglielmotto et al., 2010). Interestingly, we show that Fenchol treatment had no significant impacts on the lifespan of wild-type N2 worms (Figure 4A), while it significantly increased the survival/lifespan in worms overexpressing human Aβ (CL2006 and CL4176; Figures 4B,C). Fenchol treatment significantly reduced Aβ accumulation in worms overexpressing human Aβ (Figures 4D,E). These results suggest that the Fenchol treatment prolonged the lifespan of worms by reducing the Aβ-induced neurotoxicity. Further, Fenchol treatment decreased paralytic attacks in worms overexpressing human Aβ, compared to their non-treated controls (Figure 4F). Worms overexpressing human Aβ (CL2355) treated with Fenchol also exhibited significantly higher memory behavior during the chemotaxis assay compared to their non-Aβ expressing control worms (CL2122; Figure 4G). Overall, these results suggest that Fenchol treatment reduced neuronal cell death by reducing Aβ-accumulation, which in turn improved cognitive function and improved organism survival.

### Fenchol Increases Proteasome Activity to Reduce Aβ Accumulation

**Figure 5:**
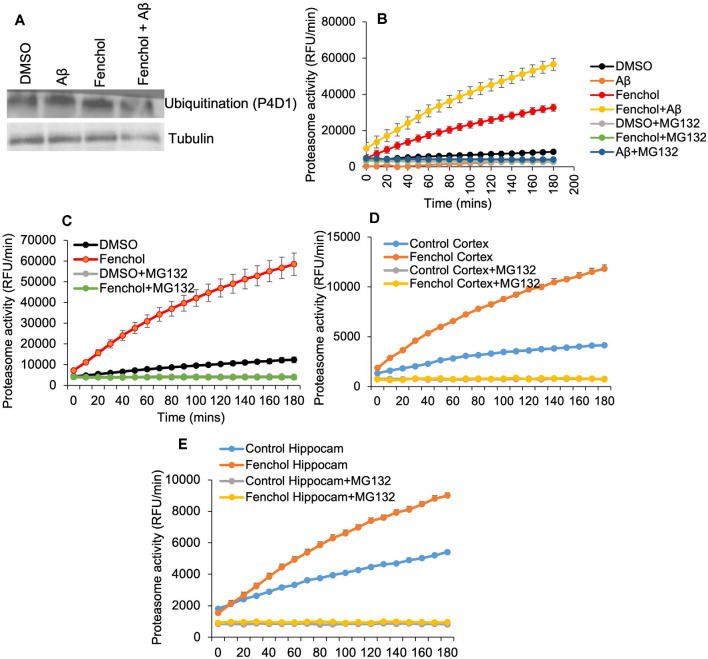
Fenchol treatment increased proteasome activity, without impacting protein ubiquitination. **(A)** Total protein ubiquitination in Western blots with anti-P4D1 antibody was not impacted by Fenchol treatment. **(B,C)** Proteasome activity was significantly increased in Fenchol treated SK-N-SH cells **(B)** and *C. elegans* **(C)**. Proteasome activity was higher in Fenchol treated SK-N-SH cells that were also treated with Aβ. Interestingly, Fenchol treatment also increased proteasome activity in APP/PS1 mice cortex **(D)** and hippocampus **(E)** regions. Data are the mean and standard error of means from three independent experiments done in triplicate.

So far we have demonstrated that Fenchol protects neuronal cells by reducing Aβ accumulation. However, the mechanisms were not known. We show that the Fenchol treatment did not change protein ubiquitinylation in the SK-N-SH cells (Figure 5A), suggesting that the reduction in Aβ accumulation with Fenchol treatment was not mediated through ubiquitin-dependent protein degradation. Interestingly, Fenchol treatment significantly increased the proteasomal activity in both SK-N-SH cells and *C. elegans* (Figures 5B,C), as well as in the cortex and hippocampus of our AD mouse model (APP/PS1; Figures 5D,E). These results indicate that Fenchol treatment reduces and clears Aβ accumulation by increasing proteasomal activity in the neuronal cells, thus reducing the neurotoxicity in these cells.

### Fenchol Reduces Cellular Senescence in Neuronal Cells

**Figure 6:**
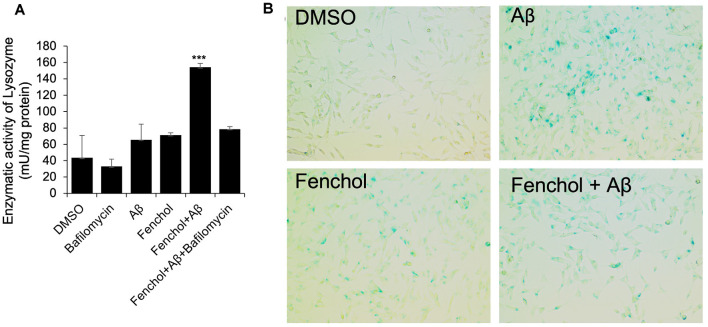
Fenchol treatment increased lysosomal activity (autophagy) **(A)** and reduced senescence **(B)** in SK-N-SH cells. Data are from three independent experiments done in triplicate and presented as the mean and standard error of means. ***P < 0.001.

To further demonstrate the mechanism of Fenchol on neuronal cell survival, we show that the Fenchol treatment significantly increased the lysosomal activity in the neuronal cells, compared to their controls (Figure 6A). This finding suggests that Fenchol increases autophagy in the neuronal cells and, thus, might be protecting these cells from Aβ-induced neurotoxicity. We also observed that the Aβ-treated cells showed a higher rate of senescence (indicated by β-galactosidase activity staining), while Fenchol treatment significantly reduced the number of senescent neuronal cells (Figure 6B). These results suggest that the mechanism of Fenchol includes improved autophagy, reduced senescence in neurons (thus, possible protection from neuronal cell death), and amelioration of AD-like pathologies in the brain.

## Discussion

Emerging evidence indicates that abnormalities in the gut microbiota and its metabolites contribute to AD pathology; however, the mechanism(s) and the precise targets by which microbiota sensing mechanisms can be manipulated to ameliorate AD pathology remain elusive. Here, we demonstrated that the inhibition of microbiota-derived SCFA signaling (FFAR2 signaling) contributes to the enhancement of Aβ-induced neurotoxicity and AD pathology. We also screened >144,000 natural compounds and upon further comprehensive validation for FFAR2 stimulating activities, found Fenchol to be a potential FFAR2 signaling agonist that exhibited protective effects against AD pathology using *in vitro*, *ex-vivo*, and *in vivo* models.

FFAR2 is abundantly expressed in a myriad of gastrointestinal tract cells as well as various other cell types, including neuronal cells. However, their function in neuronal cells is not well studied. Here, we demonstrated that the FFAR2 gene is abundantly expressed in the human neuronal cells (SK-N-SH). FFAR2 expression has also been reported in other genomics databases of human and mice brains (Atlas, 2021; Allen Brain Map, 2021); however, these analyses are done in whole brain tissues, which consist of a mixture of neurons and glial cells. Thus, the expression of FFAR2 in these types of cells individually remains unknown. Our studies demonstrated that neuronal cells express FFAR2, suggesting it might play a significant role in neuron proliferation, survival, and other functions. Our studies further confirm the importance of FFAR2 signaling in neurons and also establish SK-N-SH cells as a model to study this signaling. SCFAs, like acetate and butyrate, are natural activators of FFAR2, but they are primarily produced by microbiota in the gut, and their capability to reach the brain remains debated. Thus, natural compounds that can activate FFAR2 can be easily adapted to modulate this signaling. Therefore, we performed a large-scale in-silico screening of >144,000 natural compounds to select compounds that strongly bind to the active site of FFAR2. We selected the top 15 compounds and further validated their biological efficacy using wet-lab experiments. We used a screening strategy for finding the potential FFAR2 activators from these selected compounds, based on the knowledge that FFAR2 is a type of inhibitory G (Gi/o) protein-coupled receptor, and its activation initiates a signaling cascade which results in an intracellular increase in phosphorylation of ERK1/2 along with a reduction in cAMP and elevation in calcium (Mishra et al., 2020). Thus, we used phospho-ERK1/2 activation (phosphorylation) as primary screening for the 15 selected natural compounds and a reduction in cAMP and an increase in calcium levels as secondary screening measures. Interestingly, Fenchol was found to induce FFAR2 the most in human neuronal cells. Fenchol is a common ingredient found in edible plants like basil, grapes, mustard, aster, and alpinia speciosa root oil (Bleyer et al., 1991; Singh et al., 2015). It is also abundant in lavender oil, heartwood, and eucalyptus and is commonly used in perfumes (Zheljazkov et al., 2013; Maghsoodlou et al., 2015; Ibrahim et al., 2017). However, its role in modulating signaling pathways remains largely unknown. Here, for the first time, we have demonstrated its FFAR2 activation potential, which can reduce Aβ-induced neurotoxicity.

Accumulation of Aβ in the neurons is a hallmark of AD pathology and Aβ-induced neurotoxicity is an important mechanism by which cognitive function and AD pathology prevail. Protecting neurons from Aβ-induced cytotoxicity is an important target to prevent AD. FFAR2 inhibition increased the cytotoxic effects of Aβ on neuronal cells, suggesting that activation of FFAR2 signaling may be neuroprotective. Interestingly, Fenchol treatment protected the Aβ-induced neuronal toxicity in SK-N-SH cells; these effects were FFAR2-dependent, suggesting that Fenchol is a natural compound that can protect Aβ-induced neuronal death by activating FFAR2 signaling. However, the precise mechanisms of Fenchol in Aβ-induced neuronal cell death remain unknown. SCFAs reduce the formation of toxic Aβ aggregates (Ho et al., 2018), and Fenchol may also interfere with the formation of such toxic Aβ aggregates, causing neuronal toxicity—our future studies will test these possibilities.

*C. elegans* is an emerging model for several age-related conditions, including AD-like phenotypes (Luo et al., 2009). Several mutations, such as overexpression of human Aβ proteins, are used to develop AD-like neurodegenerative pathologies in *C. elegans* (Godini et al., 2019). For example, the CL2006 strain overexpresses human Aβ proteins, while the CL4176 strain overexpresses human Aβ in a temperature-sensitive manner (i.e., the worms grow and live normally at 16°C but overexpress Aβ and develop paralysis due to substantial neuronal death at 22°C). Additionally, the CL2355 strain overexpresses human Aβ when grown at 22°C and develops paralysis, but grows normally at 16°C (Ahmad and Ebert, 2020). CL2355 is a good model for measuring AD-like chemotactic cognition/memory behaviors compared to the CL2122 strain, which shows normal chemotaxis (cognition/memory; He et al., 2017). Interestingly, Fenchol treatment significantly increased the lifespan in the CL2006 and CL4176 strains and reduced Aβ accumulation. Fenchol also reduced paralysis in CL4176 worms, suggesting that Fenchol reduced neurotoxicity-mediated paralysis induced by Aβ accumulation. We also show that Fenchol treatment protected from a decline in chemotaxis/cognitive function of CL2355 worms, suggesting that Fenchol prevents an Aβ-induced decline in cognitive function. Altogether, these results demonstrate that Fenchol treatment significantly reduced Aβ-induced AD-like pathology in cells and *C. elegans*.

Further, the accumulation of Aβ in the brain and/or neurons is due to reduced proteolysis, which results in a decline in protein degradation or clearance (Saido and Leissring, 2012). Ubiquitination is a common protein degradation mechanism in which older/non-functional proteins get ubiquitinylated and degraded in proteasomes (Buoso et al., 2012; Vilchez et al., 2014). Fenchol significantly reduced Aβ accumulation in neuronal cells and *C. elegans*, but we found that this reduction was not due to increased ubiquitination. By investigating alternative mechanisms of Aβ reduction, we found that Fenchol significantly increased the lysosomal activity in SK-N-SH cells, Aβ overexpressing *C. elegans*, and the cortex and hippocampus of APP/PS1 mice. These findings suggest that Fenchol reduced Aβ accumulation by increasing its clearance *via* lysosomal activity and also demonstrate that the biological functions of Fenchol are conserved in worms, mice, and human cells. Lysosomal degradation is an important phenomenon of autophagy, which contributes to the efficient clearance of protein aggregates like Aβ (Lim and Yue, 2015). Reductions in lysosomal activity or the rate of autophagy are linked with increased senescence and AD pathology (Saido and Leissring, 2012; Wei et al., 2016; Curtis, 2019; Zhang et al., 2019). As expected, we show that Aβ treatment to SK-N-SH cells increased the number of senescent cells, while Fenchol significantly reduced Aβ-induced senescence in neuronal cells.

Although *C. elegans* have conserved human/mammalian neuronal pathways (Chase and Koelle, 2007; Koelle, 2018), the orthologs of FFAR2 signaling in humans vs. *C. elegans* are not well studied. Our preliminary analyses demonstrated that Galanin-like G-protein coupled receptor npr-9 has 24.01% identity with 94% query coverage in NCBI’s- BlastP analyses of FFAR2 in *C. elegans* vs. humans (Supplementary Figure S3). Further, Prosite analyses confirmed that both human FFAR2 and *C. elegans* Galanin belong to the G-protein coupled receptor family. These findings suggest that the mechanism of action of Fenchol in *C. elegans* may be through FFAR2 orthologs like npr-9; however, further comprehensive studies are needed to confirm these hypotheses. Alternatively, because many terpenoids are known to mediate Aβ-toxicity in worms, Fenchol may also act in *C. elegans* through the Nrf-2 pathway (Dostal and Link, 2010); however, these mechanisms need to be validated and established through further comprehensive studies.

## Conclusions

**Figure 7:**
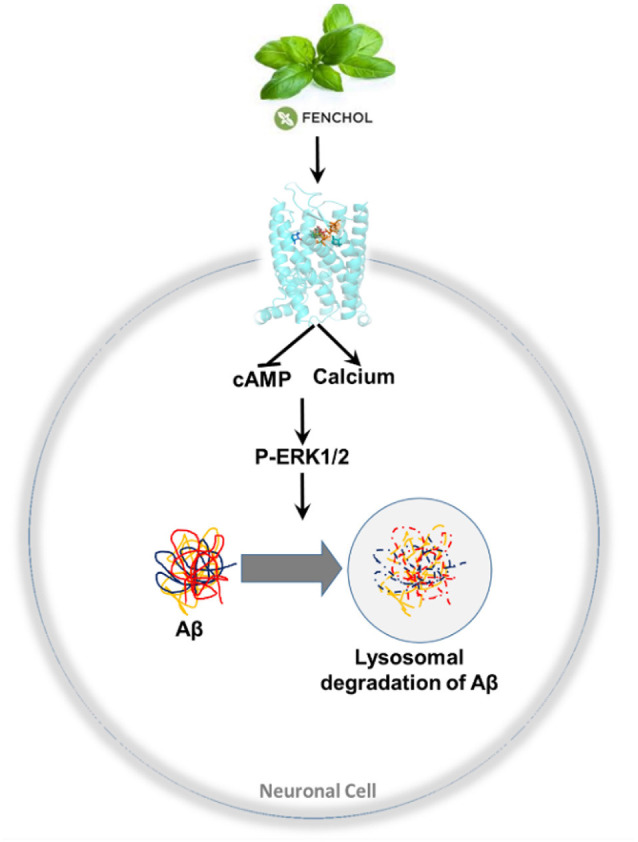
A purported model of findings in this study. Fenchol (commonly present in basil plant/leaves) activates FFAR2 signaling by increasing intracellular calcium and reducing cAMP, thus activating pERK1/2 phosphorylation, which in turn increases Aβ clearance by activating lysosomal degradation in neuronal cells. These results indicate that the activation of FFAR2 by Fenchol reduces AD-like pathology in neurons.

We demonstrated that inhibition of a microbiota sensing mechanism, FFAR2 signaling, exacerbates Aβ-induced neuronal toxicity. Thus, we screened a large-scale library of >144,000 natural compounds and discovered Fenchol as a potent activator of FFAR2 signaling. Fenchol is a common natural compound abundantly present in edible plants like basil and showed strong neuroprotective effects, which may ameliorate AD pathology. Mechanistically, Fenchol improved Aβ clearance by increasing lysosomal activities, which, in turn, reduced cellular senescence in neuronal cells. Overall, our results demonstrate that the inhibition of FFAR2 signaling exacerbates Aβ-induced neurotoxicity, while its activation by Fenchol reverses these abnormalities, and suggests that potential FFAR2 activators like Fenchol can prevent AD progression (Figure 7).

## Data Availability Statement

The original contributions presented in the study are included in the article/Supplementary Material, further inquiries can be directed to the corresponding author.

## Ethics Statement

The animal studies were reviewed and approved by Institutional Animal Care and Use Committee (IACUC) at Wake Forest School of Medicine and IACUC at University of South Florida.

## Author Contributions

AR performed experiments and analyzed data. PK, SM, and SS performed and helped bioinformatics analyses. SJ and BM contributed significantly in data interpretations, writing manuscripts, and intellectual discussions. HY conceived the idea, supervised the study, helped in data interpretations, developed and wrote the manuscript, and revised drafts of manuscripts. All authors contributed to the article and approved the submitted version.

## Conflict of Interest

The authors declare that the research was conducted in the absence of any commercial or financial relationships that could be construed as a potential conflict of interest.

## Publisher’s Note

All claims expressed in this article are solely those of the authors and do not necessarily represent those of their affiliated organizations, or those of the publisher, the editors and the reviewers. Any product that may be evaluated in this article, or claim that may be made by its manufacturer, is not guaranteed or endorsed by the publisher.
