# Supplementary material for: Activation of Microbiota Sensing – Free Fatty Acid Receptor 2 Signaling Ameliorates Amyloid-β Induced Neurotoxicity by Modulating Proteolysis-Senescence Axis
Source: Front Aging Neurosci. 2021 Oct 5;13:735933. doi: 10.3389/fnagi.2021.735933 (PMC8544178; doi:10.3389/fnagi.2021.735933)
Supplement: Supplementary file 1 [file Data_Sheet_1.PDF]

Supplementary Table S1. List of natural compound libraries used for screening.

S.No.	Name of Libraries	No. of Compounds
1	AfroDb Natural Products	1,008
2	AnalytiCon Discovery NP	20,000
3	Herbal Ingredients In-Vivo Metabolism	1,465
4	Herbal Ingredients Targets	9,862
5	IBScreen NP	68,000
6	Indofine Natural Products	20,000
7	Nubbe Natural Products	2,147
8	Specs Natural Products	800
9	TCM Database @ Taiwan	20,000
10	NPACT Database	1,574
**Total**		**144,856**

Supplementary table S2. Table of binding energies for top 15 compounds.

S.No.	ZINC ID	Compound Name	[the energy of the lowest energy conformation]
1	ZINC00157548	Norpseudoephedrine hydrochloride	-6.39
2	ZINC95099135	7-hydroxycamphene	-6.18
3	ZINC00074836	Ephedrine	-6.01
4	ZINC00388198	Octopamine hydrochloride	-5.94
5	ZINC01597139	(±)-Carvomenthol	-5.86
6	ZINC59587245	4-Carene	-5.8
7	ZINC01081099	FENCHOL	-5.73
8	ZINC02034811	3-Pinanone	-5.73
9	ZINC00403588	Synephrine	-5.73
10	ZINC00968099	Borneol	-5.7
11	ZINC00968029	Darwinol	-5.7
12	ZINC02040990	Beta-terpineol	-5.69
13	ZINC00403588	Synephrine	-5.68
14	ZINC00967571	(+)-Fenchone	-5.68
15	ZINC03581377	L-Leucinamide hydrochloride	-5.68

Supplementary Table S3a. Agonist and antagonist probability prediction of top 15 compounds interaction with human Ffar2 homology model

**S. No.**	**Compounds**	**H-Bonding Residues Human_FFAR2**	**Probability as Agonist Ser86, Tyr90, His140, Ile145, Val179, Arg180, Leu183, Tyr238, His242, Arg255 Interaction with both Arg180 & Arg255 - Agonist**	**Probability as Antagonist Ser86, Tyr90, His140, Ile145, Val179, Arg180, Leu183, Tyr238, His242, Arg255 Interaction with either Arg180 or Arg255 - Antagonist**
1	175 (Acetate)	**His242**, Tyr238	Potential Agonist	NA
2	ZINC00895132 (Butyrate)	Tyr238	Potential Agonist	NA
3	ZINC000118616157 (CatPB)	Gln166, Tyr238, **Arg255**, Lys65, Gln148, Ser86, Ser256	NA	Potential Antagonist
4	ZINC00157548 (Norpseudoephedrine hydrocholoride)	Tyr238, **His242**, Leu183, Gln148, Cys164, Glu166, Ser86, Thr85, Glu68	20%	0%
5	ZINC95099135 (7-Hydroxycamphene)	Thr85, Glu166, Ser86, Gln148, **Tyr238**, Glu68, **His242**	30%	0%
6	ZINC00074836 (Ephedrine)	Thr85, Gln148, Glu166, Ser86, Glu68, Cys164, **His242**	10%	0%
7	ZINC00388198 (Octopamine hydrocholride)	Leu183, **Tyr238**, His242, Asn239, Gln148, Cys164, Glu166, **Ser86**, Glu68, Thr85,	70%	0%
8	ZINC01597139 (Carvomenthol)	**Ser86**, Gln148, **Tyr238**, Glu166, Thr85, Glu68, **Arg255**	20%	60%
9	ZINC59587245 (4-Carene)	All Hydrophobic	0%	0%
10	ZINC01081099 (Fenchol)	Glu166, **Ser86**, **His242**, Gln148, **Tyr238**	50%	0%
11	ZINC02034811 (3-Pinanone)	**Ser86**, **Arg255**, Gln148	30%	70%
12	ZINC00403588 (Synephrine)	Leu183, Tyr238, **His242**, Gln148, Glu166, **Ser86**, Glu68, Thr85, **Tyr238**, **Arg255**	20%	70%
13	ZINC00968099 (Borneol)	**Ser86**, Glu166, Glu68, **Tyr238**, **Arg255**	10%	80%
14	ZINC00968029 (Darwinol)	Ser263, **Ser86**, Gln148, Glu166, Glu68, Cys164	20%	0%
15	ZINC02040990 (Beta-terpineol)	Cys184, Thr85, Cys164, **Tyr238**, Gln148, Glu166, **Arg255**, Glu68, **Ser86**	30%	60%
16	ZINC00967571 (Fenchone)	Gln148, **Tyr238**, Lys65	10%	0%
17	ZINC03581377 (Leucinamide hydrocholoride)	Ser263, Ile10, Lys65, Val259, Glu68, Gln148	0%	0%

Supplementary table S3b. Agonist and antagonist probability prediction of top 15 compounds interaction with Mice Ffar2 homology model

S.No.	Compounds	H-Bonding Residues Mice_FFAR2	Probability as Agonist Ser86, Tyr90, His140, Ile145, Val179, Arg180, Leu183, Tyr238, His242, Arg255 Interaction with both Arg180 & Arg255 - Agonist	Probability as Antagonist Ser86, Tyr90, His140, Ile145, Val179, Arg180, Leu183, Tyr238, His242, Arg255 Interaction with either Arg180 & Arg255 - Antagonist
1	175 (Acetate)	Tyr90, **Arg180**, Gln148, Asn265, Arg65, Gln148, Tyr238	Potential Agonist	NA
2	ZINC00895132 (Butyrate)	Tyr238, Arg65, **Arg180**, Tyr90, Gln170	Potential Agonist	NA
3	ZINC000118616157 (CatPB)	Asn151, Asn167, Arg255, Gln172, Pe168	NA	Potential Antagonist
4	ZINC00157548 (Norpseudoephedrine hydrocholoride)	Cys164, Val147, Asn167, Thr169, Arg65, Gln148, Val81, Thr85, **Arg255**, Leu60, Glu320	10%	60%
5	ZINC95099135 (7-Hydroxycamphene)	Thr85, Gln323, Gln170, Glu320, Thr169, Gln318	0%	70%
6	ZINC00074836 (Ephedrine)	**Arg255**, Val81, Thr85, Glu320, Ile146, Val147, Asn167	0%	70%
7	ZINC00388198 (Octopamine hydrocholride)	Glu68, Trp75, Glu166, Val147, Asn167, Thr169, Leu60, Thr85, Glu320, Gln323, Ala319, Ile146 Gln170, **Arg255**, Gln155, Leu60, Val81, Thr85	0%	70%
8	ZINC01597139 (Carvomenthol)	Arg255, Asn167, Thr169, Val81, Thr85, Leu60, Phe64	0%	60%
9	ZINC59587245 (4-Carene)	All hydrophobic	0%	0%
10	ZINC01081099 (Fenchol)	Glu320, Thr85	0%	0%
11	ZINC02034811 (3-Pinanone)	**Arg255**, Thr169, Gln170	0%	80%
12	ZINC00403588 (Synephrine)	Glu68, Trp75, Tyr90, Gln148, Cys164, Tyr238, Asn167, Phe168, Arg255, Ile146, Glu170, Val81, Thr85, Val147, Asn167, Thr169, Gln155	0%	80%
13	ZINC00968099 (Borneol)	Thr169, Gln170, Val81, Thr85, **Arg255**, Leu60, Glu320	0%	80%
14	ZINC00968029 (Darwinol)	Gln148, Ala319, Gln323, Glu320, Leu60, Val81, Thr85	0%	0%
15	ZINC02040990 (Beta-terpineol)	Glu166, Tyr90, Gln148, Tyr238, Val156, **Arg255**, Ile146, Val147	0%	60%
16	ZINC00967571 (Fenchone)	Arg255, Gln170	0%	50%
17	ZINC03581377 (Leucinamide hydrocholoride)	Arg65, Tyr90, Glu166, Val147, Asn167, Thr169, Gln170, Glu320, **Arg255**, Asn151	0%	50%

Supplementary Figure S1. In-silico Modeling and interaction data from human and mice Ffar2 homology modeling

Acetate interaction with human and mouse Ffar2 homology modeling

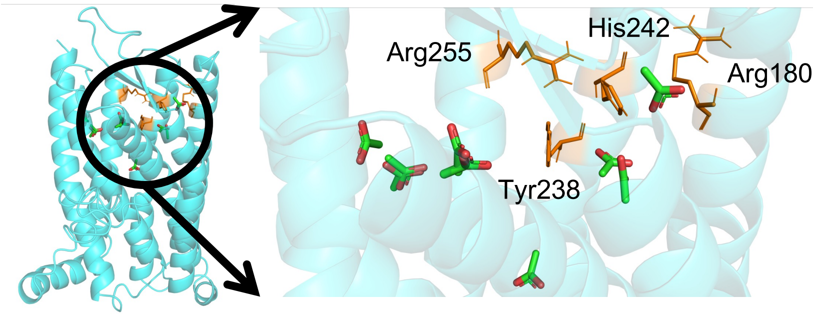

Acetate Interaction with human Ffar2

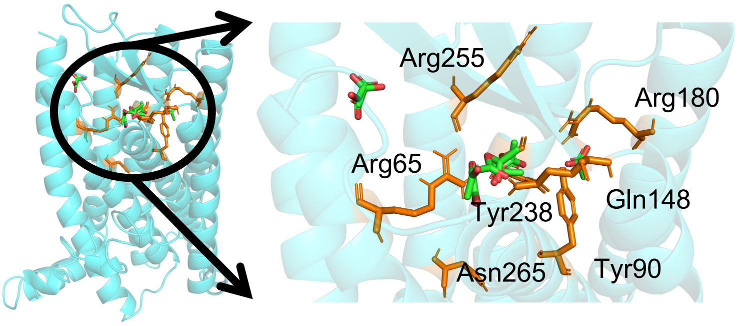

Acetate Interaction with mouse Ffar2

Butyrate interaction with human and mouse Ffar2 homology modeling

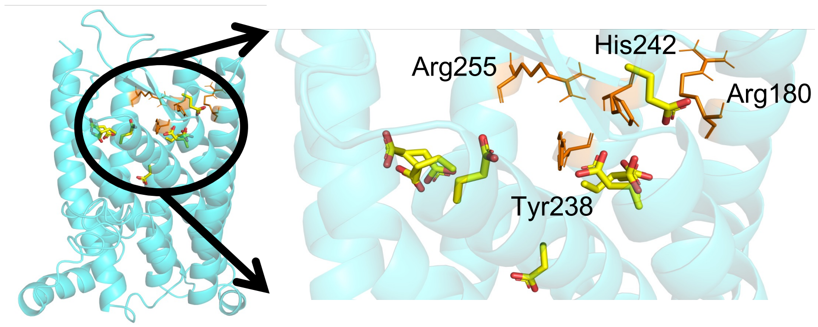

Butyrate Interaction with human Ffar2

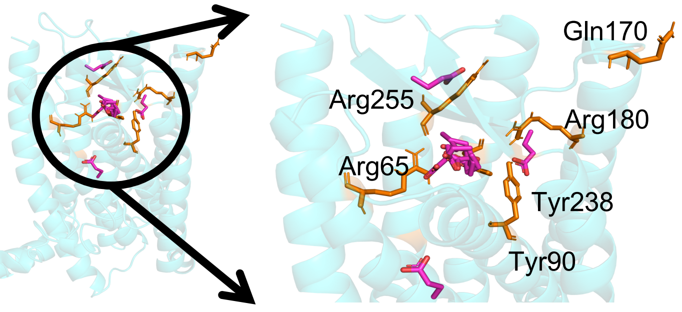

Butyrate Interaction with mouse Ffar2

Norpsedoephedrine hydrochloride (ZINC00157548) interaction with human and mouse Ffar2 homology modeling

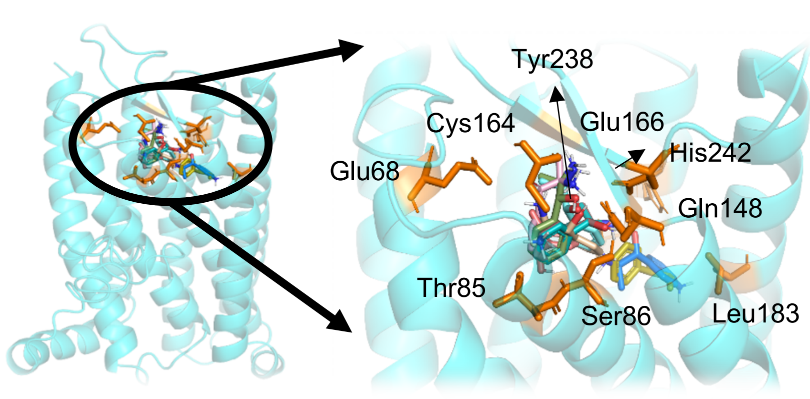

ZINC00157548 interaction with human Ffar2

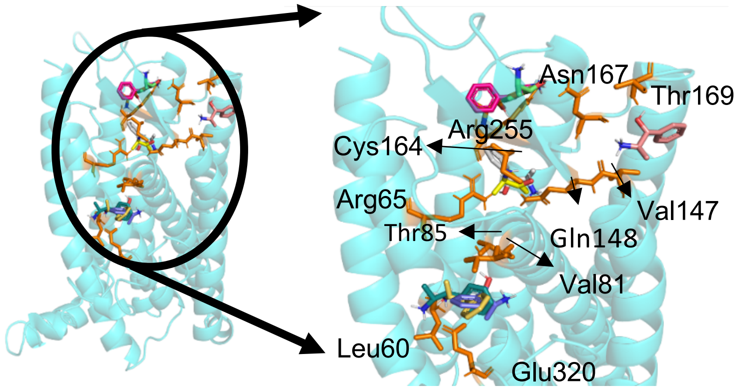

ZINC00157548 interaction with mouse Ffar2

7-Hydroxycamphene (ZINC95099135) interaction with human and mouse Ffar2 homology modeling

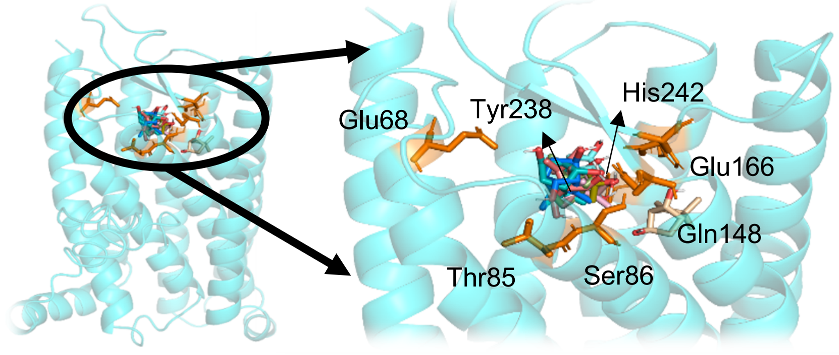

ZINC95099135 interaction with human Ffar2

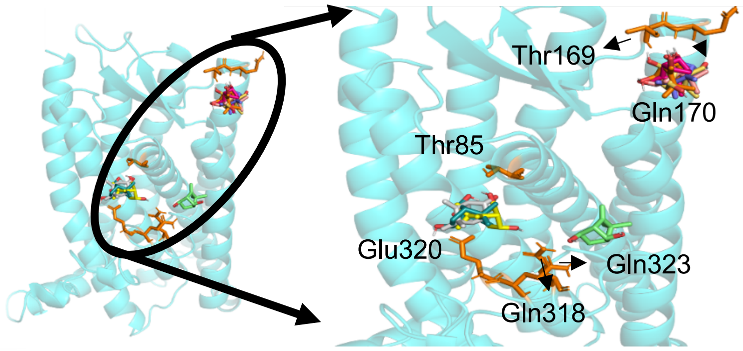

ZINC95099135 interaction with mouse Ffar2

Ephedrine (ZINC00074836) interaction with human and mouse Ffar2 homology modeling

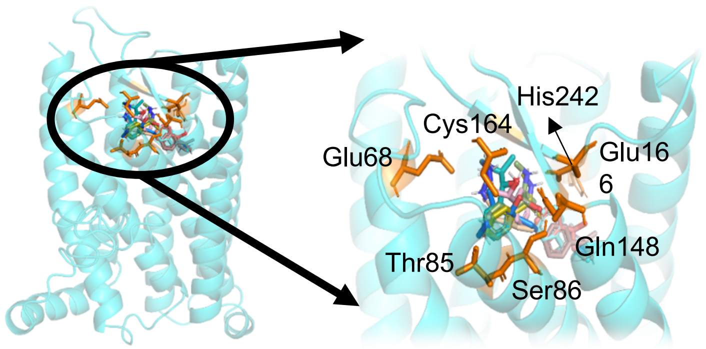

ZINC00074836 interaction with human Ffar2

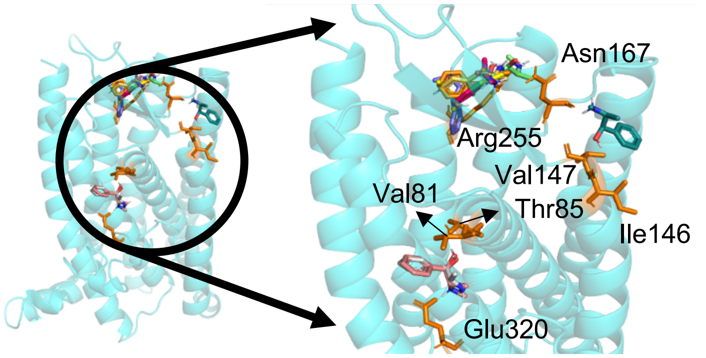

ZINC00074836 interaction with mouse Ffar2

Octopamine Hydrochloride (ZINC00388198) interaction with human and mouse Ffar2 homology modeling

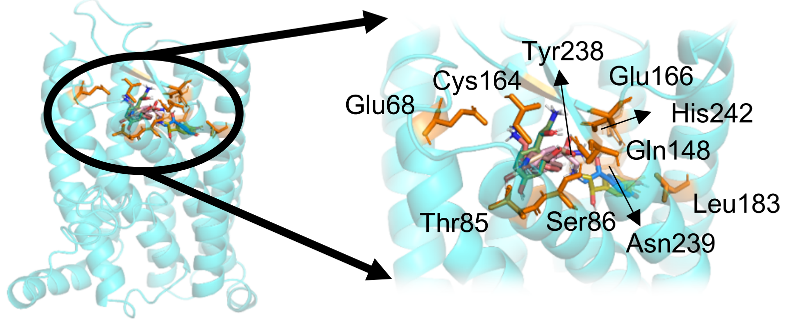

ZINC00388198 interaction with human Ffar2

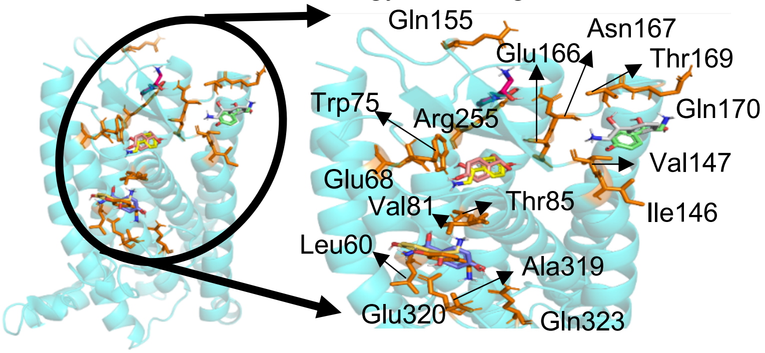

ZINC00388198 interaction with mouse Ffar2

Carvomenthol (ZINC01597139) interaction with human and mouse Ffar2 homology modeling

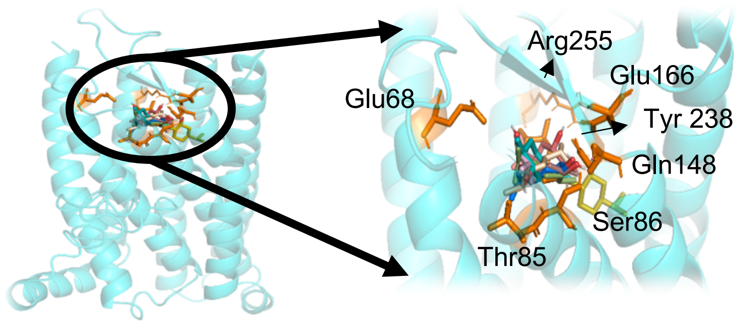

ZINC01597139 interaction with human Ffar2

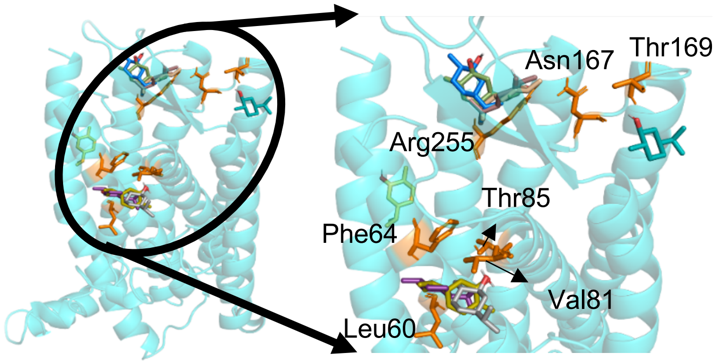

ZINC01597139 interaction with mouse Ffar2

4-Carene (ZINC59587245) interaction with human and mouse Ffar2 homology modeling

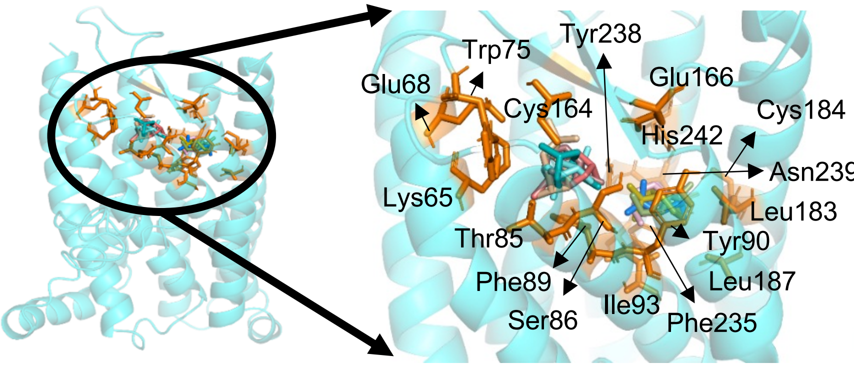

ZINC01597139 interaction with human Ffar2

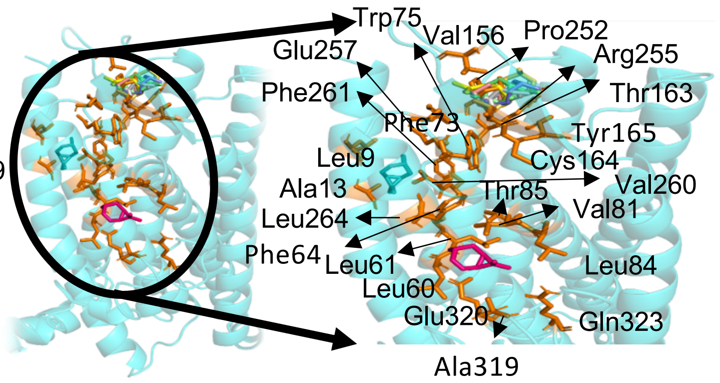

ZINC01597139 interaction with mouse Ffar2

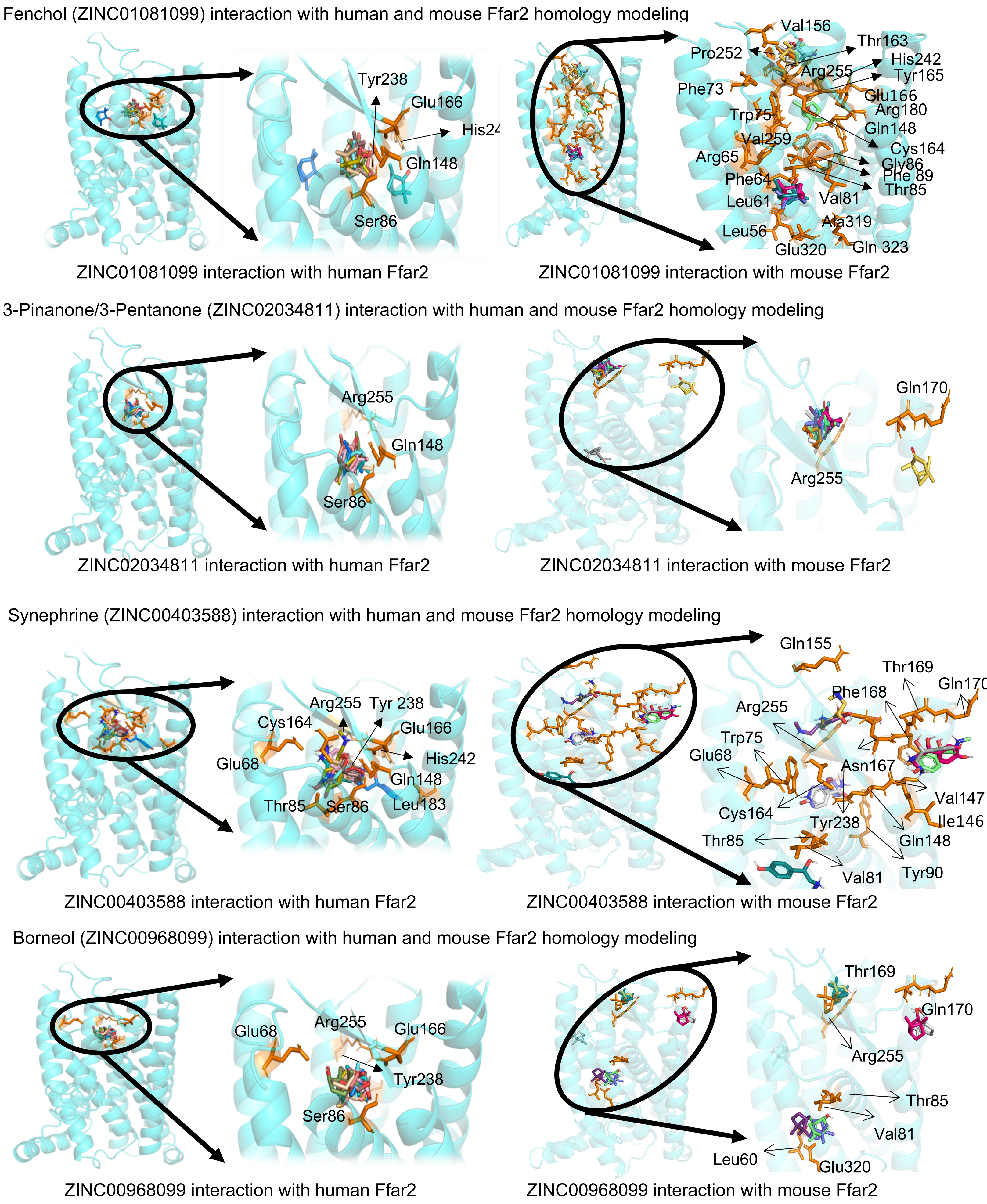

Fenchol (ZINC01081099) interaction with human and mouse Ffar2 homology modeling
Tyr238
Glu166
His24
Gln148
Ser86
ZINC01081099 interaction with human Ffar2
Val156
Thr163
Pro252
His242
Arg255
Tyr165
Phe73
Glu166
Arg180
Trp75
Gln148
Val259
Cys164
Arg65
Gly86
Phe 89
Phe64
Thr85
Leu61
Val81
Ala319
Leu56
Gln 323
Glu320
ZINC01081099 interaction with mouse Ffar2
3-Pinanone/3-Pentanone (ZINC02034811) interaction with human and mouse Ffar2 homology modeling
Arg255
Gln148
Ser86
ZINC02034811 interaction with human Ffar2
Gln170
Arg255
ZINC02034811 interaction with mouse Ffar2
Synephrine (ZINC00403588) interaction with human and mouse Ffar2 homology modeling
Arg255
Tyr 238
Cys164
Glu166
His242
Glu68
Gln148
Thr85
Ser86
Leu183
ZINC00403588 interaction with human Ffar2
Gln155
Thr169
Phe168
Arg255
Trp75
Glu68
Asn167
Val147
Cys164
Tyr238
Ile146
Thr85
Gln148
Val81
Tyr90
ZINC00403588 interaction with mouse Ffar2
Borneol (ZINC00968099) interaction with human and mouse Ffar2 homology modeling
Glu68
Arg255
Glu166
Tyr238
Ser86
ZINC00968099 interaction with human Ffar2
Thr169
Gln170
Arg255
Thr85
Val81
Leu60
Glu320
ZINC00968099 interaction with mouse Ffar2

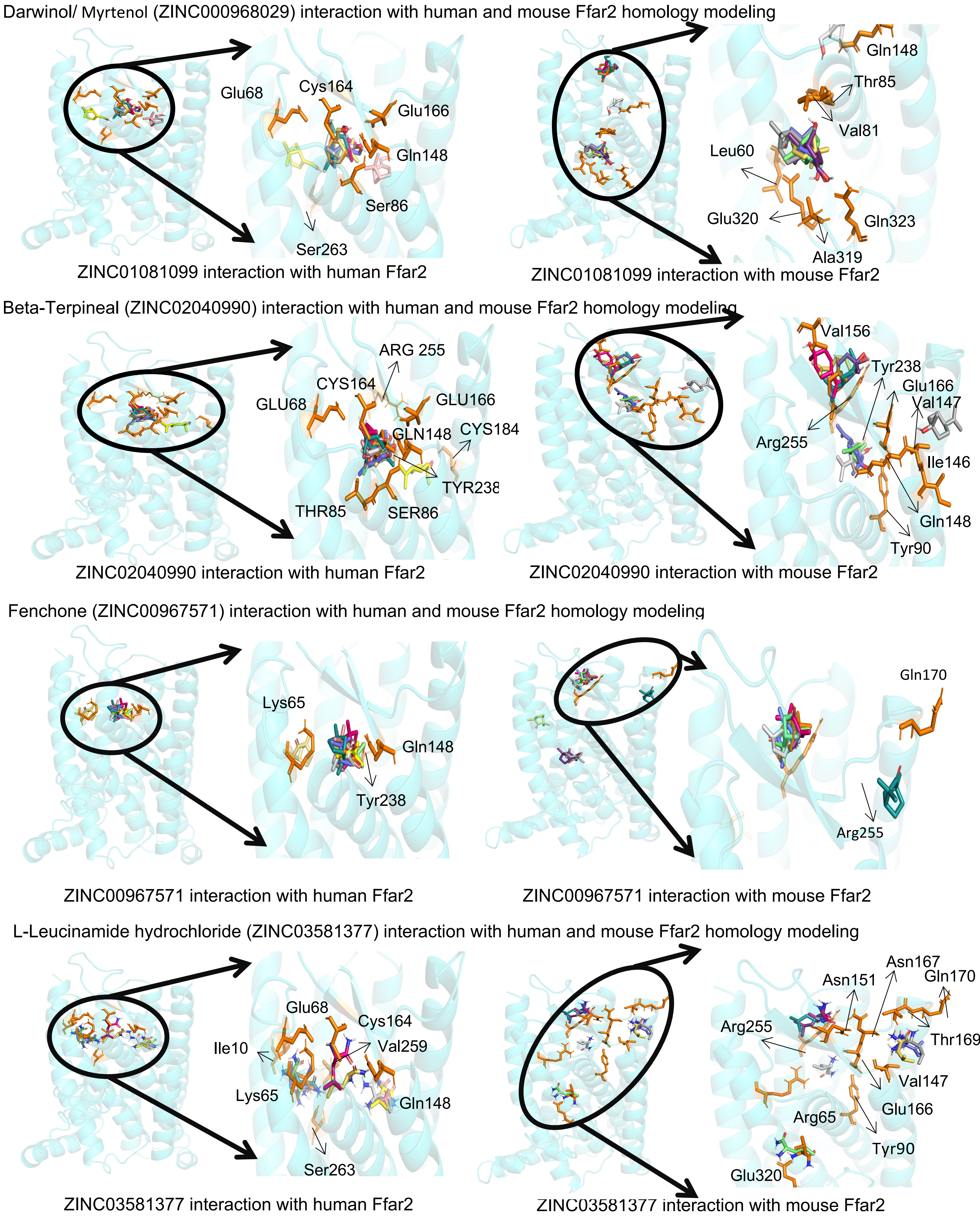

Darwinol/ Myrtenol (ZINC000968029) interaction with human and mouse Ffar2 homology modeling
Glu68
Cys164
Glu166
Gln148
Ser86
Ser263
ZINC01081099 interaction with human Ffar2
Gln148
Thr85
Val81
Leu60
Glu320
Gln323
Ala319
ZINC01081099 interaction with mouse Ffar2
Beta-Terpineal (ZINC02040990) interaction with human and mouse Ffar2 homology modeling
ARG 255
CYS164
GLU68
GLU166
GLN148
CYS184
TYR238
THR85
SER86
ZINC02040990 interaction with human Ffar2
Val156
Tyr238
Glu166
Val147
Arg255
Ile146
Gln148
Tyr90
ZINC02040990 interaction with mouse Ffar2
Fenchone (ZINC00967571) interaction with human and mouse Ffar2 homology modeling
Lys65
Gln148
Tyr238
Gln170
Arg255
ZINC00967571 interaction with human Ffar2
ZINC00967571 interaction with mouse Ffar2
L-Leucinamide hydrochloride (ZINC03581377) interaction with human and mouse Ffar2 homology modeling
Glu68
Cys164
Ile10
Val259
Lys65
Gln148
Ser263
ZINC03581377 interaction with human Ffar2
Asn167
Asn151
Gln170
Arg255
Thr169
Val147
Glu166
Arg65
Tyr90
Glu320
ZINC03581377 interaction with mouse Ffar2

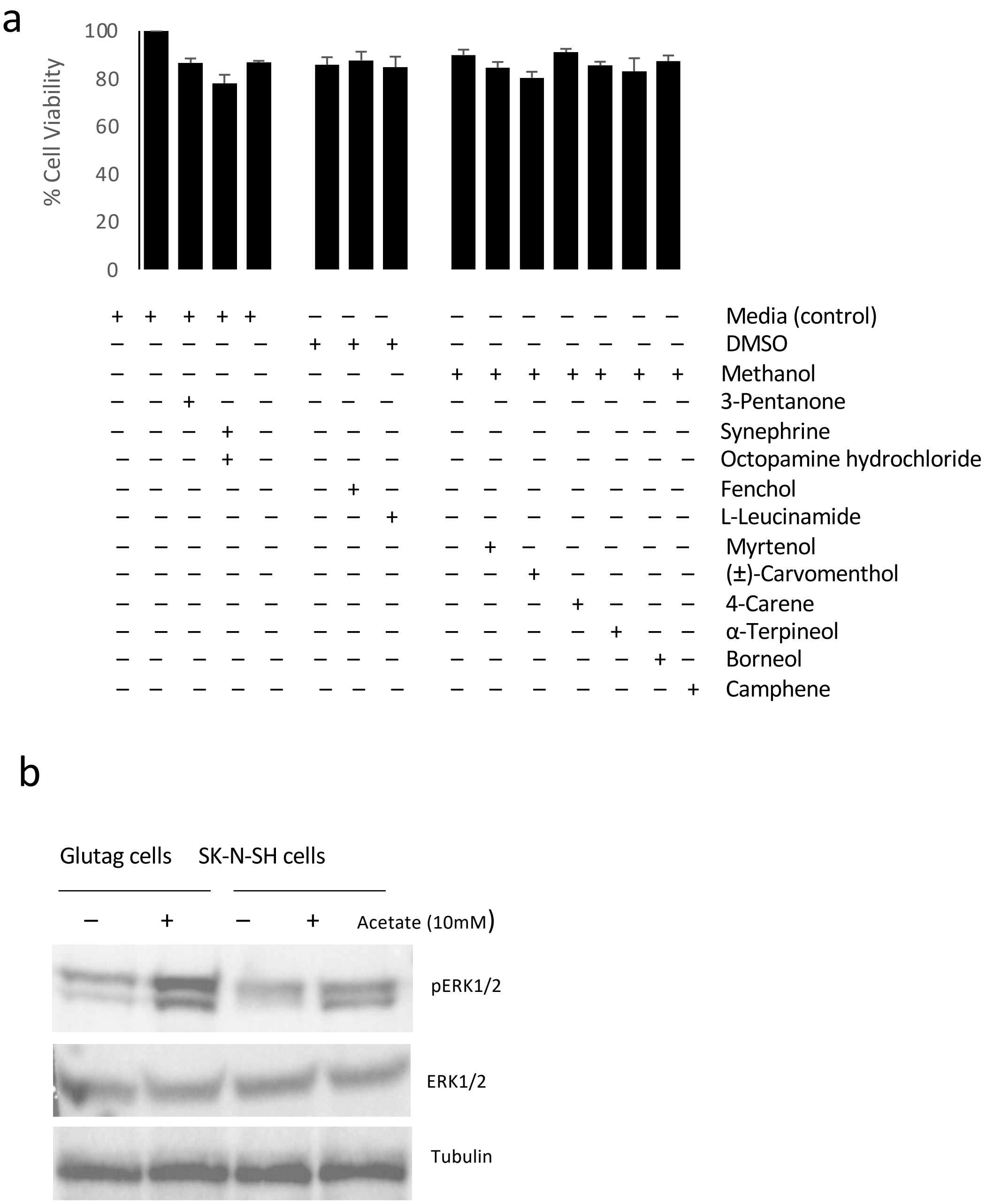

Figure S2. Effect of selected compounds on cell viability of neural cell. a) SK-N-SH cell lines were treated with 10μM of each compounds at 37 °C for 24 h. b) Expression of phpsphoERK1/2 protein in Glutag and SK-N-SH cells. Both cell lines were treated with 10mM acetate at 37 °C for 30 min.

Fasta sequence of FFAR2 Human: Length:330

```
>sp|O15552|FFAR2_HUMAN Free fatty acid receptor 2 OS=Homo sapiens OX=9606
GN=FFAR2 PE=1 SV=1
MLPDWKSSLILMAYIIIFLTGLPANLLALRAFVGRIRQPQPAPVHILLLSLTLADLLLLL
LLPFKIIEAASNFRWYLPKVVCALTSFGFYSSIYCSTWLLAGISIERYLGVAFPVQYKLS
RRPLYGVIAALVAWVMSFGHCTIVIIVQYLNTTEQVRSGNEITCYENFTDNQLDVVLPVR
LELCLVLFFIPMAVTIFCYWRFVWIMLSQPLVGAQRRRRAVGLAVVTLLNFLVCFGPYNV
SHLVGYHQRKSPWWRSIAVVFSSLNASLDPLLFYFSSSVVRRAFGRGLQVLRNQGSSLLG
RRGKDTAEGTNEDRGVGQGEGMPSSDFTTE
```

BLASTp output

Descriptions | Graphic Summary | Alignments | Taxonomy

Sequences producing significant alignments   Download   New Select columns   Show 100

select all   29 sequences selected   GenPept   Graphics   Distance tree of results   Multiple alignment   New MSA Viewer

Description	Scientific Name	Max Score	Total Score	Query Cover	E value	Per. Ident	Acc. Len	Accession
G_PROTEIN_RECEP_F1_2 domain-containing protein [Caenorhabditis elegans]	Caenorhabditis elegans	57.4	57.4	94%	4e-09	24.01%	397	NP_001024264.1
G_PROTEIN_RECEP_F1_2 domain-containing protein [Caenorhabditis elegans]	Caenorhabditis elegans	53.5	53.5	83%	8e-08	23.62%	433	NP_001024265.1
G_PROTEIN_RECEP_F1_2 domain-containing protein [Caenorhabditis elegans]	Caenorhabditis elegans	43.1	43.1	72%	2e-04	20.47%	402	NP_509725.2
G_PROTEIN_RECEP_F1_2 domain-containing protein [Caenorhabditis elegans]	Caenorhabditis elegans	39.3	39.3	52%	0.004	22.16%	763	NP_505884.1
G_PROTEIN_RECEP_F1_2 domain-containing protein [Caenorhabditis elegans]	Caenorhabditis elegans	39.3	39.3	52%	0.004	22.16%	761	NP_001360566.1
G_PROTEIN_RECEP_F1_2 domain-containing protein [Caenorhabditis elegans]	Caenorhabditis elegans	43.1	43.1	49%	2e-04	25.30%	457	NP_508816.1
G_PROTEIN_RECEP_F1_2 domain-containing protein [Caenorhabditis elegans]	Caenorhabditis elegans	50.4	50.4	47%	1e-06	24.85%	564	NP_001024804.1
G_PROTEIN_RECEP_F1_2 domain-containing protein [Caenorhabditis elegans]	Caenorhabditis elegans	50.4	50.4	47%	1e-06	26.71%	592	NP_001024805.1
G_PROTEIN_RECEP_F1_2 domain-containing protein [Caenorhabditis elegans]	Caenorhabditis elegans	49.7	49.7	47%	1e-06	24.85%	485	NP_001041275.1
G_PROTEIN_RECEP_F1_2 domain-containing protein [Caenorhabditis elegans]	Caenorhabditis elegans	35.4	35.4	47%	0.044	21.64%	408	NP_741432.3
G_PROTEIN_RECEP_F1_2 domain-containing protein [Caenorhabditis elegans]	Caenorhabditis elegans	35.4	35.4	47%	0.045	21.64%	407	NP_001368379.1
G_PROTEIN_RECEP_F1_2 domain-containing protein [Caenorhabditis elegans]	Caenorhabditis elegans	35.4	35.4	47%	0.046	21.64%	433	NP_001360504.1
G_PROTEIN_RECEP_F1_2 domain-containing protein [Caenorhabditis elegans]	Caenorhabditis elegans	35.4	35.4	47%	0.047	21.64%	414	NP_001293726.1
G_PROTEIN_RECEP_F1_2 domain-containing protein [Caenorhabditis elegans]	Caenorhabditis elegans	35.4	35.4	47%	0.047	21.64%	427	NP_001360506.1
G_PROTEIN_RECEP_F1_2 domain-containing protein [Caenorhabditis elegans]	Caenorhabditis elegans	35.4	35.4	47%	0.049	21.64%	402	NP_001370376.1

BLASTp output – 94% query coverage

Motif search using Prosite: https://prosite.expasy.org/PS00237
Both proteins FFAR2 and Galanin belongs to GPCR family with the given motif

```
FFAR2_HUMAN/24-273
ANLLALRAFVGRIRQpqPAPVHILLLSLTLADLLLLLLLPFKIIEAASNFRWYLPKVVCA
Galanin-like G-protein coupled receptor npr-9 [Caenorhabditis elegans]
UniProt id: Q23497
NPR9_CAEEL/46-329
NFFQHTSAYCSVWTLTLMALDRYLAVVYPVESMTLRTPRNTVIALCFIYIIIIASQIPVG
```

Figure S3. Similarity search of FFAR2 ortholog in C. elegans versus human FFAR2 protein.
